# Examining the role of psychological factors in the relationship between sleep problems and suicide

**DOI:** 10.1016/j.cpr.2017.03.009

**Published:** 2017-06

**Authors:** D. Littlewood, S.D. Kyle, D. Pratt, S. Peters, P. Gooding

**Affiliations:** a Division of Psychology & Mental Health, School of Health Sciences, University of Manchester, UK; b Manchester Academic Health Science Centre, University of Manchester, UK; c Sleep and Circadian Neuroscience Institute, Nuffield Department of Clinical Neurosciences, University of Oxford, UK; d Centre for Health Psychology, University of Manchester, UK

**Keywords:** Suicide, Suicidal ideation, Suicidal behaviour, Sleep, Systematic review

## Abstract

We sought to conduct the first systematic review of empirical evidence investigating the role of psychological factors in the relationship between sleep problems and suicidal thoughts and behaviours. Twelve studies were identified which examined psychological factors grouped into four categories of cognitive appraisals, psychosocial factors, emotion regulation strategies, and risk behaviours. Although there was substantial heterogeneity across studies with respect to measurement, sampling, and analysis, preliminary evidence indicated that negative cognitive appraisals, perceived social isolation, and unhelpful emotion regulation strategies may contribute to the association between sleep problems and suicidal thoughts and behaviours. Given that findings in this area are currently restricted to studies with cross-sectional designs, the directionality of the interrelationships between these psychological factors, sleep problems and suicidality, remains unclear. We integrate the findings of our review with contemporary psychological models of suicidal behaviour to develop a clear research agenda. Identified pathways should now be tested with longitudinal and experimental designs. In addition, a more thorough investigation of the complexities of sleep, psychological factors, and suicidal thoughts and behaviours is crucial for the development of targeted psychological interventions.

## Highlights

• Systematic review of the role of psychological factors in sleep/suicide relationships
• Review findings integrated with suicide theory to define a clear research agenda.
• Reliance on cross-sectional designs limits interpretation of directionality of pathways.
• Identified pathways should be investigated with longitudinal and experimental designs.

## 1 Introduction

“*The best bridge between despair and hope is a good night*'*s sleep*”.

E. Joseph Cossman

Each year approximately 800,000 lives worldwide are lost to suicide, equating to one death every 40 s (World Health Organisation, 2014). In the US, the most recent estimates of suicide deaths indicate that there were 13.4 suicides per 100,000 people in 2014 (Centers for Disease Control and Prevention, 2015), and in the UK this estimate was 10.8 suicides per 100,000 (Office for National Statistics, 2016). Interventions to prevent suicide are important, not only to the individual at risk of suicide, but also to the many people who are likely to be affected in the event of an individual's death. For example, those bereaved by suicide experience elevated levels of depression (Séguin, Lesage, & Kiely, 1995), substance use (Brent, Melhem, Donohoe, & Walker, 2009), and suicidal behaviours (Pitman, Osborn, King, & Erlangsen, 2014).

Suicidal thoughts and behaviours have been conceptualised to lie on a continuum whereby those who experience suicidal thoughts may progress to make suicide plans and then, subsequently, make an attempt or die by suicide (Johnson et al., 2008, Tarrier et al., 2013, ten Have et al., 2009). Indeed, prevalence data from a study of 17 countries estimated that 33.6% of individuals who experience suicidal thoughts and feelings will subsequently develop a suicide plan, and 56% of those with a plan will make a suicide attempt (Nock et al., 2008). Therefore, identifying and intervening at the start of this trajectory is imperative to developing effective suicide prevention strategies.

### 1.1 Sleep problems as a modifiable risk factor for suicidality

Research into suicide has identified a number of clinical and sociodemographic risk factors which may trigger and maintain suicidal thoughts and behaviours, such as, mental health problems, sleep problems, unemployment, gender, and age (Bernal et al., 2007, Borges et al., 2010, Nock et al., 2008, Pigeon et al., 2012, Qin et al., 2000). Although many of the identified risk factors are impossible or difficult to change (e.g., gender, unemployment), sleep patterns can be effectively modified using existing psychological interventions (Wu, Appleman, Salazar, & Ong, 2015). Furthermore, recent findings indicate that cognitive-behavioural therapy for insomnia not only benefits sleep, but may also reduce levels of suicidal ideation (Trockel, Karlin, Taylor, Brown, & Manber, 2015). Moreover, reducing risk of suicide via treatment of sleep problems may be more acceptable to those individuals who are reluctant to seek treatment for mental health problems due to perceptions of stigma, for example (Bernert, Iwata, Kim, Moscowitz, & Horn, 2015).

Sleep problems are commonly experienced by individuals with mental health problems, such as depression and post-traumatic stress disorder. Consequently, it is important to understand the interrelationships between specific mental health problems, sleep problems and suicidal behaviour. For instance, one possible explanation of the sleep/suicide relationship is that sleep operates indirectly to increase suicide risk, because sleep problems increase the likelihood of depression (Baglioni et al., 2011) and depression, in turn, increases the risk of suicidal thoughts and behaviours (Harris & Barraclough, 1997). Findings from studies examining the interrelations between depression, sleep problems and suicidality are mixed, with divergent patterns of results emerging from studies examining insomnia (Bryan et al., 2015, Nadorff et al., 2014, Nadorff et al., 2013, Ribeiro et al., 2012). However, research examining nightmares has consistently shown an association between nightmares and suicidal thoughts and behaviours, independent of the effects of depression (Pigeon et al., 2012). This indicates that the role of depression in the sleep/suicide relationship varies dependent on the specific type of sleep problem experienced. Therefore, it is important to determine which other factors may influence the relationship between suicidal ideations and acts, and different types of sleep problems. Furthermore, given the high prevalence of sleep problems in clinical and non-clinical populations (Roth et al., 2006), it would be prudent to establish the possible transdiagnostic, psychological mechanisms which account for the sleep/suicide relationship.

### 1.2 Role of psychological processes in the association between sleep problems and suicidality

It is plausible that psychological factors may play a role in the mechanisms underlying the relationships between sleep problems and suicidal thoughts and behaviours. This is because the presence of sociodemographic risk factors alone do not account for the complexity and variance within suicidal pathways (Gooding et al., 2015, Johnson et al., 2008, O'Connor and Nock, 2014, Panagioti et al., 2015, Tarrier et al., 2013). Understanding psychological mechanisms that underpin suicidal pathways is crucial to the development of clinical interventions to prevent subsequent suicide attempts and deaths (Johnson et al., 2008, O'Connor and Nock, 2014, Tarrier et al., 2013). Previous reviews have proposed possible psychological processes and mechanisms which may influence the sleep/suicide relationship (McCall and Black, 2013, Winsper and Tang, 2014, Woznica et al., 2015). However, no previous review has systematically evaluated and critically appraised the empirical evidence base relating to the role of specific psychological processes and mechanisms in the sleep/suicide relationship. This was the main aim of the current review.

Psychological theories of suicide provide a framework for understanding the way in which a combination of the vulnerabilities produced from sociodemographic risk factors may interact with cognitive and psychological processes to account for the development of suicidal thoughts and behaviours (Johnson et al., 2008, O'Connor and Nock, 2014, Tarrier et al., 2013). For example, psychological factors, such as hopelessness, have been shown to further elevate risk of suicide in individuals who also reported sociodemographic risk factors, such as living alone and unemployment (Steeg et al., 2016). Practically, these theoretical frameworks are important in the development of clinical interventions. The Medical Research Council guidelines on developing interventions highlight the importance of a coherent theoretical basis for the development of optimally effective interventions (Craig et al., 2008). In recognition of this, the second aim of the current review was to integrate psychological theories of suicide with the review findings to outline the role of psychological factors in the relationship between sleep problems and suicidal thoughts and behaviours, from which hypotheses for future work can be generated and tested.

## 2 Method

### 2.1 Search strategy

In order to be comprehensive and maximally inclusive, studies were sought which aimed to examine the relationship between sleep quality and/or sleep disorders, suicidal thoughts and behaviours, and a psychological process or factor. To aid comprehension, the term ‘suicidality’ will be used for instances where both suicidal thoughts and behaviours were examined.

Four electronic databases (EMBASE, Medline, PsycINFO, Web of Science) were searched up to July 2016, using a combination of Medical Subject Heading (MeSH) terms and text words for suicide and sleep. Given that there are differences in the indexing of MeSH terms used by the different databases, a full list of all identified terms is detailed in Appendix A. Filters were used to limit search results to those published in journals in English.

Hand searches were conducted in two stages. First, the reference sections within all papers identified for inclusion within the review were scrutinised for omissions. Second, the reference sections of existing review and position papers on the relationship between sleep and suicide were also checked for omissions (Agargun and Besiroglu, 2005, Bernert and Joiner, 2007, Bernert et al., 2015, Bernert and Nadorff, 2015, Liu and Buysse, 2006, Malik et al., 2014, McCall and Black, 2013, Norra et al., 2011, Pigeon and Caine, 2010, Pigeon et al., 2012, Singareddy and Balon, 2001, Winsper and Tang, 2014, Woznica et al., 2015). Furthermore, the publication history of prominent researchers actively investigating the sleep suicide relationship, were reviewed for additional papers which could be included (i.e., Agargun, Bernert, McCall, Nadorff, Pigeon and Hochard).

### 2.2 Eligibility criteria

Studies were included in the review based on the following four eligibility criteria:

1. An empirical study (quantitative or qualitative) published in a peer reviewed journal in the English language.
2. Reported original empirical findings (i.e., reviews, practice recommendations, comments, replies, letters, opinion/position papers, practice guidelines, conference abstracts or theses were excluded).
3. Aimed to examine the role of psychological factor(s) in explaining the relationship between sleep problems and suicidal thoughts or behaviours. In quantitative work this is most clearly exemplified by pathway, mediation or moderation analyses. In qualitative studies this approach is most clearly illustrated by topic guides and analyses that make specific reference to the relationship between sleep and suicide.
4. Included a measure of any type of suicidal thoughts, plans, risk, behaviours, and deaths (papers examining self-injury without suicide intent were not included); an objective or subjective assessment of any type of sleep problem (e.g., trouble getting to sleep at night, nightmares, hypersomnia, insomnia); and a measure of a psychological factor. For the purposes of the review, variables were deemed to be a psychological factor if they represented cognitions (e.g., memory, attention, executive function, information-processing), emotions (e.g., happiness, sadness, fear), metacognitions (e.g., appraisals of sociocognitive emotional factors, hopelessness, defeat, entrapment), psychosocial factors (e.g., loneliness, social isolation) or risk behaviours (e.g., aggression, impulsivity). Papers which measured the presence of mental health problem (e.g., psychosis, major depressive disorder) together with sleep problems and suicidality, but with no measurement of psychological factors were excluded.

In order to optimise inclusion for this review, we used no restrictions pertaining to the age of the sample (e.g., adolescents, working age adults, elderly individuals).

### 2.3 Management of search outcomes and study eligibility screening

**Fig. 1:**
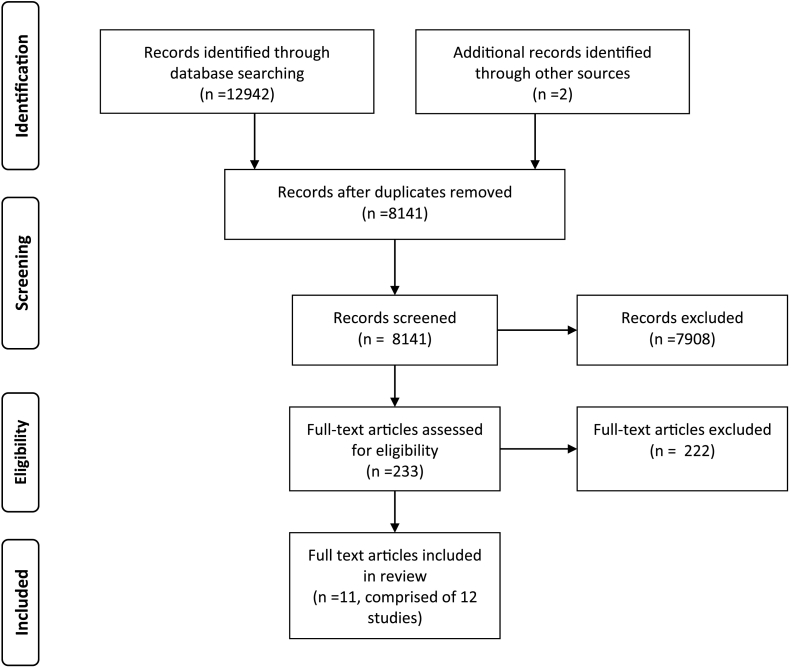
PRISMA flow diagram illustrating the processes of literature searches and screening.

This systematic review was conducted with guidance from the PRISMA 2009 statement (Moher, Liberati, Tetzlaff, & Altman, 2009). Fig. 1 provides an overview of the search and screening processes. The first author (DL) screened the search results against the identified eligibility criteria. In addition, the last author (PG) screened 10% of the identified studies, to provide a measure of the reliability of the screening process, with any disagreements resolved through discussion between the two authors.

### 2.4 Quality appraisal

Critical appraisal of the methodological quality of the identified studies was conducted independently by the first (DL) and last (PG) authors. Discrepancies in ratings were resolved through discussion between the two authors. It was considered appropriate to evaluate quality specifically based on criteria pertinent to the research designs employed within the identified studies. Consequently, six criteria were adapted from the modified Newcastle-Ottawa Quality Assessment Scale for cross-sectional studies (Herzog et al., 2013) and the Walsh and Downe (2006) scale for appraising qualitative studies. Relevant criterion from both scales were selected to permit assessment against the same key criteria pertaining to quality of the reported research methodology and analysis for both the qualitative and quantitative studies identified within this search (see Appendix B). Total quality ratings for each study are included in Table 1, with 0–3 indicating low quality, 4–6 moderate quality, and 7–9 high quality. Formally evaluating the quality of the identified studies provided an additional indication of the strength of the evidence, alongside the extent to which findings converged.

**Table 1:** Summary of studies which have examined the relationship between sleep, suicidality and psychological factors.

Study	Sample	Design	Sleep variable	Suicide variable	Psychological variable	Analysis	Main finding	Quality score
Bozzay et al. (2016)	483 healthy undergraduate students *M*age = 20.4 (3.87), 100% female	Cross-sectional	Insomnia (ISI)	Suicidal thoughts (ASIQ)	Hopelessness (BHS); Fatigue (MFI); appraised social-problem solving (SPSI-R-SF)	Path analysis	Hopelessness, social problem-solving and fatigue partially explained the relationship between insomnia and suicidal thoughts, independent of depressive symptoms. Higher depressive symptoms moderated the relationships between social problem-solving and hopelessness, to amplify suicidal thoughts.	7
Chu et al. (2016)	552 healthy undergraduate students *M*age = 21.53 (2.25), 74.5% female	Cross-sectional	Insomnia (ISI)	Suicidal thoughts (DSI-SS)	Thwarted belongingness (INQ)	Multiple linear regression and mediation analysis	Thwarted belongingness significantly mediated the relationship between insomnia severity and suicidal thoughts.	7
Golding et al. (2015)	167 older adults aged 55 +. *M*age = 60.64 (4.94), 74.3% female	Cross-sectional	Insomnia (ISI); Insomnia duration (If you have an insomnia problem, how long have you had it for?); Nightmares (DDNSI); Nightmares duration (single item from DDNSI)	Suicidality (SBQ)	Acquired capability (ACSS-FAD); Perceived burdensomeness and thwarted belongingness (INQ)	Multiple linear regression	Duration of nightmares was associated with suicidality, independent of acquired capability for suicide, perceived burdensomeness, thwarted belongingness, current insomnia symptoms, and current nightmares. However, duration of insomnia symptoms was not significantly associated with suicidality independent of acquired capability for suicide, perceived burdensomeness, thwarted belongingness, current insomnia symptoms, current nightmares and duration of nightmares.	7
Hochard et al. (2016)	540 adults *M*age = 24.2 (7.9), 74.3% female	Cross-sectional	Insomnia (ISI); Nightmares (DDNSI)	Suicidal thoughts (DSI-SS)	Acquired capability (mod-DSHI) Entrapment (Entrapment scale)	Multiple linear regression	Acquired capability interacted with both insomnia and nightmares to significantly predict suicidal thoughts. Entrapment interacted with both insomnia and nightmares to significantly predict suicidal thoughts.	7
Littlewood, Gooding, Kyle, et al. (2016)	18 individuals with experience of depression and suicidality. *M*age = 33 (N/A), 44% female	Qualitative interviews	Sleep problems (subjective participants' narratives, PSQI, SCI, SDSQ)	Suicidality (subjective participant narrative, SSI)	Negative situational and self-appraisals, social isolation, rumination, entrapment	Thematic analysis	Sleep was perceived as contributing to suicidality via three interrelated pathways. Four key psychological processes were identified which underpin these pathways, namely, negative situational and self-appraisals, social isolation, rumination, entrapment.	8
Littlewood, Gooding, Panagioti, et al. (2016)	91 individuals with PTSD symptoms. *M*age = 28.87 (10.64), 73% female	Cross-sectional	Nightmares (sum of item 2 ratings for intensity and severity from CAPS)	Suicidality (SBQ-R)	Defeat (Defeat scale); Entrapment (Entrapment scale); Hopelessness (BHS)	Mediation analysis	There was a significant indirect pathway whereby nightmares led to defeat, which led to entrapment, then hopelessness, and finally to suicidality. The direct relationship between nightmares and suicidality remained significant.	6
McCall et al. (2013)	50 patients with depressive disorders. *Ma*ge = 49 (N/A), 72% female	Cross-sectional	Insomnia (ISI); Nightmares (DDNSI)	Suicidal thoughts (SSI)	Negative sleep-related appraisals (DBAS-16)	Mediation analysis	Insomnia was indirectly related to suicidal thoughts through nightmares and dysfunctional beliefs and attitudes about sleep.	8
Nadorff et al. (2014) - Study 1	747 healthy undergraduate students. *M*age = 18.9 (1.4), 57% female	Cross-sectional	Insomnia (ISI); Nightmares (DDNSI)	Suicidality (SBQ-R, reported a previous suicide attempt)	Acquired capability (ACSS-FAD); Perceived burdensomeness and thwarted belongingness (INQ)	Multiple linear regression and logistic regression	Insomnia was not related to suicidality when acquired capability for suicide, perceived burdensomeness and thwarted belongingness were controlled for. Nightmares were significantly related to suicidality after controlling for acquired capability, perceived burdensomeness and thwarted belongingness.	8
Nadorff et al. (2014) - Study 2	604 healthy undergraduate students. *M*age = 20.72 (4.15), 79.5% female	Cross-sectional	Insomnia (ISI); Nightmares (DDNSI)	Suicidal behaviour (series of questions adapted from the L-SASI)	Acquired capability (ACSS-FAD); Perceived burdensomeness and thwarted belongingness (INQ)	Multiple linear regression	Both insomnia and nightmares were related to suicidal behaviour, after controlling for acquired capability for suicide, perceived burdensomeness and thwarted belongingness.	8
Weis et al. (2015)	460 healthy community-dwelling young adults. *M*age = 25.6 (3.1), 74.1% female	Cross-sectional	Sleep quality (PSQI)	Suicidality (SBQ-R)	Emotion regulation (ERQ); rumination (subscale from the RSQ)	Structural equational modelling	Sleep problems were indirectly related to suicidality through depression severity, emotion regulation, and rumination.	5
Woosley et al. (2014)	766 healthy community-dwelling. *M*age = 53.78 (19.85), 50.8% female	Cross-sectional	Insomnia (identified against DSM-V criteria as documented in responses in 14-day sleep diary, ESS, FSS, IIS, BDI, and STAI)	Suicidal thoughts (item 9 from BDI. Transformed into a dichotomous variable of present versus absent)	Hopelessness (item 2 from BDI)	Mediation analysis	The indirect pathway by which hopelessness mediated the relationship between insomnia and suicidal thoughts was significant.	5
Zschoche and Schlarb (2015)	93 healthy adolescents. Aged 14 to 18 years, *M*age = N/A, 69.9% female	Cross-sectional	Sleep quality (PSQI)	Suicidality (PSS)	Aggressive behaviour (Aggressive behaviour subscale from the FEPAA)	Mediation analysis	Aggressive behaviour did not significantly mediate the relationship between sleep problems and suicidality.	6

Note: NA, information not available in the article. PTSD = Post-traumatic Stress Disorder. Measures abbreviations, ACSS-FAD = Acquired Capability for Suicide Scale–Fearlessness about Death; ASQI = Adult Suicide Ideation Questionnaire; BDI = Beck Depression Inventory; BHS = Beck Hopelessness Scale; SSI = Beck Scale for Suicide Ideation; CAPS = Clinician-Administered PTSD Scale for DSM-IV; DSI-SS = Depressive Symptoms Inventory–Suicidality Subscale; DDNSI = Disturbing Dreams and Nightmare Severity Index; DBAS-16 = Dysfunctional Beliefs and Attitudes Scale-brief; ERQ = Emotion Regulation Questionnaire; ESS = Epworth sleepiness scale; FSS = Fatigue Severity Scale; FEPAA = German Questionnaire for Acquiring Empathy, Prosociality, Readiness for Aggression, and Aggressive Behavior; IIS = Insomnia Impact Scale; ISI = Insomnia Severity Index; INQ = Interpersonal Needs Questionnaire; L-SASI = Lifetime Suicide Attempt Self-Injury Interview; MFI = Multidimensional Fatigue Inventory; PSS = Paykel Suicide Scale; PSQI = Pittsburgh Sleep Quality Index; RSQ = Response Style Questionnaire; SCI = Sleep Condition Index; SDSQ = Sleep Disorders Screening Questionnaire; SPSI-R-SF = Social Problem Solving Inventory–Revised: Short Form; STAI-Y = State-Trait Anxiety Inventory-form Y; SBQ = Suicidal Behaviors Questionnaire; SBQ-R = Suicidal Behaviors Questionnaire Revised.

## 3 Results

### 3.1 Study characteristics

The literature search yielded twelve original research studies that investigated the role of psychological variables in relation to the association between sleep problems and suicidality (see Table 1). These were reported in 11 papers. Five studies were based on student or adolescent samples (Bozzay et al., 2016, Chu et al., 2016, Nadorff et al., 2014^1^; Zschoche & Schlarb, 2015), four studies included healthy community-dwelling participants (Golding et al., 2015, Hochard et al., 2016, Weis et al., 2015, Woosley et al., 2014) and the remaining three studies were conducted with clinical populations (Littlewood et al., 2016, Littlewood et al., 2016, McCall et al., 2013). All studies adopted a quantitative cross-sectional design (n = 11), with the exception of a single qualitative study in which an inductive thematic analysis was performed (Littlewood, Gooding, Kyle, et al., 2016).

Three types of sleep-related variables were assessed with subjective measures, which were sleep quality (Littlewood et al., 2016, Weis et al., 2015, Zschoche and Schlarb, 2015), nightmares (Golding et al., 2015, Hochard et al., 2016, Littlewood et al., 2016, Littlewood et al., 2016, Nadorff et al., 2014), and insomnia (Bozzay et al., 2016, Chu et al., 2016, Golding et al., 2015, Hochard et al., 2016, McCall et al., 2013, Nadorff et al., 2014, Woosley et al., 2014). However, none of the identified studies utilised objective measures of sleep. Furthermore, suicidality was operationalised across these twelve studies using eight different assessment tools, namely, The Adult Suicide Ideation Questionnaire (Reynolds, 1991), The Beck Scale for Suicide Ideation (Beck, Kovacs, & Weissman, 1979), The Suicidal Behaviors Questionnaire (Linehan, 1981), The Suicidal Behaviors Questionnaire-Revised (Osman et al., 2001), The Paykel Suicide Scale (Paykel, Myers, Lindenthal, & Tanner, 1974), the Depressive Symptoms Inventory–Suicidality Subscale (Metalsky & Joiner, 1997), the Lifetime Suicide Attempt Self-Injury Interview (Linehan & Comtois, 1996) and item 9 from the Beck Depression Inventory (Beck & Steer, 1987). There was much heterogeneity in the psychological factors investigated, which have been categorised broadly into four groups comprising cognitive appraisals, psychosocial factors, emotion regulation strategies, and risk behaviours. Eight studies investigated two or more psychological variables and were evaluated within each relevant category (Bozzay et al., 2016, Golding et al., 2015, Hochard et al., 2016, Littlewood et al., 2016, Littlewood et al., 2016, Nadorff et al., 2014, Weis et al., 2015). Finally, all quantitative studies conducted preliminary analyses to examine the relationship between sleep problems and suicidality (Bozzay et al., 2016, Chu et al., 2016, Golding et al., 2015, Hochard et al., 2016, Littlewood et al., 2016, McCall et al., 2013, Nadorff et al., 2014, Weis et al., 2015, Woosley et al., 2014, Zschoche and Schlarb, 2015). Thereafter, a range of analytical approaches were used to examine the influence of psychological factors on this relationship.

### 3.2 Study quality

Quality ratings for the studies included in this review ranged between five and eight (M = 6.8), indicating that they were of moderate to high quality. Individual ratings for each study are included in Table 1. One of the key criticisms of wider research that has examined the relationship between sleep problems and suicidality, is the failure to include a measurement of depression (Bernert, Kim, et al., 2015). This is important given that sleep problems are highly prevalent in depression (Soehner et al., 2014, Tsuno et al., 2005), and that there is a strong association between depression and suicidal thoughts and behaviours (Hawton et al., 2013, Oquendo et al., 2006, Tarrier et al., 2013). This is reflected in depression measures which generally include items relating to both sleep and suicidal thoughts and behaviours. Therefore, the role of depression should be quantified with the aim of ensuring that identified psychological factors are contributing to the sleep/suicide relationship, rather than the sleep/depression or depression/suicide relationships. It is noteworthy that ten of the quantitative studies identified in this review accounted for the role of depression, albeit with different approaches. Golding et al. (2015) focused specifically on controlling for the impact of anhedonic symptoms of depression on analyses. The remaining nine quantitative studies either included depressive symptoms as an additional mediating variable (Weis et al., 2015, Zschoche and Schlarb, 2015) or took steps to statistically control for the impact of depressive symptoms or comorbid diagnoses of depression, within the main analyses (Bozzay et al., 2016, Hochard et al., 2016, Littlewood et al., 2016, McCall et al., 2013, Nadorff et al., 2014, Woosley et al., 2014). The qualitative study sought to explicitly examine the interrelationships between depressive symptoms, sleep problems, and suicidality (Littlewood, Gooding, Kyle, et al., 2016). Finally, one quantitative study opted not to include a measure of depression within analyses based on the rationale that having accounted for variance in suicidality explained by depression, the remaining variance is largely error variance (Chu et al., 2016).

### 3.3 Which psychological processes account for the association between sleep problems and suicidality?

#### 3.3.1 Cognitive appraisals

##### 3.3.1.1 Hopelessness

Three studies (Bozzay et al., 2016, Littlewood et al., 2016, Woosley et al., 2014) examined hopelessness within the context of the sleep/suicide relationship, although in different ways. First, using data from a cross-sectional study of a healthy community-dwelling sample, Woosley et al. (2014) tested a mediational pathway whereby the relationship between insomnia and suicidal thoughts was postulated to operate via hopelessness. Results indicated that hopelessness significantly mediated the relationship between insomnia and suicidal thoughts. One prominent strength of this study was the comprehensive measurement of insomnia against DSM-V criteria (Woosley et al., 2014). However, as the authors acknowledge, the use of a single-item to measure hopelessness is problematic because it fails to capture the multi-dimensional nature of this construct (Beck et al., 1974, Woosley et al., 2014).

Second, Littlewood and colleagues (Littlewood, Gooding, Panagioti, et al., 2016) tested a theoretically driven (Williams et al., 2005, Williams and Williams, 1997) mediational pathway, examining the relationship between intensity and severity of nightmares and suicidality using a cross-sectional design with people who had symptoms of PTSD. As predicted, intensity and severity of nightmares were associated with suicidality indirectly through three serial mediators which were defeat, entrapment, and hopelessness. Here, hopelessness was identified as the third mediator within the pathway, and was indirectly related to nightmares via defeat first and then entrapment. There were two key strengths of the analysis strategy taken by this study. First, the pattern of results remained the same when analyses were repeated in a subset of participants without comorbid symptoms of depression, which indicates that this pathway operates independent of depression. Second, in the pursuit of isolating the specific pathway between nightmares and suicidality, comorbid insomnia was included as a control variable. However, findings are limited by the operationalisation of nightmares which was measured through the summation of two-items from the Clinician-Administered PTSD Scale for DSM-IV (Littlewood, Gooding, Panagioti, et al., 2016).

Third, hopelessness was included within a four-step mediational pathway, whereby severity of insomnia symptoms was related to suicidal thoughts through fatigue, then to appraisals of social problem-solving, and finally to hopelessness (Bozzay et al., 2016). Hopelessness was posited to emerge from negative appraisals of social problem-solving, and subsequently trigger suicidal thoughts. A cross-sectional design with female undergraduate students was conducted to test the predicted pathways. Path analyses supported the hypothesis with greater severity of depressive symptoms also shown to heighten the relationships between appraisals of social problem-solving and hopelessness, and hopelessness and suicidal thoughts (Bozzay et al., 2016). A key strength of this study was the combination of psychological theories and empirical evidence to develop a conceptual framework from which the association between insomnia and suicidal thoughts could be understood. In addition, validated scales were used to measure all variables. Conclusions are limited by the exclusively female non-clinical sample. Consequently, it remains to be seen whether these pathways extend to both genders and to people who experience severe mental health problems.

##### 3.3.1.2 Negative situational and self-appraisals

To date, three studies have highlighted the role of negative situational and self-appraisals within the relationship between sleep problems and suicidal thoughts and behaviours (Bozzay et al., 2016, Littlewood et al., 2016, McCall et al., 2013). First, a cross-sectional study (McCall et al., 2013) focused on negative appraisals that specifically relate to sleep using the Dysfunctional Beliefs and Attitudes about Sleep Scale (Morin, Vallières, & Ivers, 2007). This 16-item scale consists of four subscales which assess beliefs about the consequences of insomnia; perceptions of worry and helplessness about sleep problems; expectations about sleep; and beliefs about the effects of sleep medication. McCall et al. (2013) tested a mediational pathway whereby dysfunctional beliefs and attitudes about sleep, and nightmares, were predicted to mediate the relationship between severity of insomnia symptoms and suicidal thoughts, in people with depressive disorders. Bootstrapped mediational analyses indicated that the relationship between insomnia and suicidal thoughts was mediated by dysfunctional beliefs about sleep and the frequency and intensity of nightmares. However, the specific indirect effect via dysfunctional beliefs about sleep showed a trend towards significance (95% CI: − 0.03–0.97). Methodologically, this study demonstrated high quality because it used validated scales to measure all study variables, and the determination of sample size through an a priori power calculation. Furthermore, the robust sampling strategy accounted for the possible presence of other underlying sleep disorders by excluding patients who had confirmed or suspected sleep apnea or restless leg syndrome. Future work examining negative beliefs about sleep should include supplementary mediational analyses of the subscales assessed by the Dysfunctional Beliefs and Attitudes about Sleep Scale, which may indicate if any particular type of negative sleep-related appraisals contribute to the sleep/suicide relationship.

Second, an exploratory, qualitative study examined the role of sleep in relation to suicidality, in people with experience of a major depressive episode(s) (Littlewood, Gooding, Kyle, et al., 2016). Different cognitive factors were identified in participant's narratives, including negative appraisals about current situations, and negative appraisals relating to the self. During the daytime, participants perceived that they had reduced cognitive abilities resulting from lack of sleep the previous night, which was associated with negative appraisals of self-worth. At night, situational appraisals concerning the lack of activity during night-time hours fuelled perceptions of isolation and loneliness, which perpetuated negative self-appraisals. Maximum variation sampling was used to effectively recruit participants who had experienced different types of sleep problems. Subsequently, the thematic analysis proposed core pathways which appeared to underpin the role of sleep in suicidality, rather than focusing on a specific type of sleep complaint. However, divergent findings indicate that the putative mechanisms which underpin the relationship between suicidality and nightmares, may differ to those which underpin the relationship between suicidality and insomnia (Golding et al., 2015, Nadorff et al., 2014).

Third, a cross-sectional study tested a conceptual model of the relationship between insomnia and suicidal thoughts which included appraisals of fatigue and social problem-solving ability (Bozzay et al., 2016). Perceptions of fatigue were posited to negatively impact appraisals of social problem-solving due to the perception of reduced mental resources, which consequently increases the likelihood of avoiding problems, making impulsive judgements, and making greater reasoning errors (Bozzay et al., 2016). As predicted, insomnia was related to suicidal thoughts through perceptions of fatigue, which led to negative appraisals of social problem-solving, and then led to hopelessness. Interestingly, the relationship between fatigue and social problem-solving did not vary as a function of depression severity, which indicates that negative self-appraisals may be driven by depleted energy and cognitive resources, rather than depressed mood.

##### 3.3.1.3 Defeat and entrapment

The negative effects of defeat and entrapment on suicidal thoughts and behaviours have been emphasised in numerous models of suicidal thoughts and behaviours (Johnson et al., 2008, O'Connor, 2011, Williams and Williams, 1997, Williams et al., 2005). A recent meta-analysis reported a strong effect size between perceptions of defeat and entrapment and suicidality (Siddaway, Taylor, Wood, & Schulz, 2015). Two studies included within this review examined defeat and entrapment in the context of the sleep/suicide relationship (Littlewood et al., 2016, Littlewood et al., 2016). Drawing on the Cry of Pain model of suicide (Williams and Williams, 1997, Williams et al., 2005), Littlewood, Gooding, Panagioti, et al. (2016) examined the role of perceptions of defeat, and entrapment in the relationship between nightmares and suicidality. As predicted, bootstrapped mediational analyses indicated that the relationship between nightmares and suicidality operated indirectly via three serial mediators of defeat, entrapment and hopelessness. The direct relationship between nightmares and suicidality remained significant, indicating that the outlined mediational pathways did not fully account for this relationship. Analyses were consistent when participants with comorbid depression were removed from the sample.

A subsequent qualitative study conducted by Littlewood, Gooding, Kyle, et al. (2016) sought to examine the role of sleep within suicidal pathways. Here, participants described a strong desire to use sleep to escape from the problems in their waking lives (Littlewood, Gooding, Kyle, et al., 2016). These narratives reflected perceptions of defeat, from which sleep provided an escape, as exemplified by this quote from one of the participants.

“….it feels like a blessed release that you're unconscious for, how many hours and that you're no longer thinking about your worthlessness and that you don't want to exist.”

(ID11, male)

This study highlights a pathway whereby failure to sleep appears to intensify perceptions of entrapment because an escape route involving sleep is continually prevented by insomnia.

A recent cross-sectional study in a non-clinical sample investigated the role of entrapment, but not defeat, in accounting for the sleep/suicide relationship (Hochard et al., 2016). Two types of sleep problems were examined within this study, namely, current severity of insomnia symptoms and frequency and intensity of nightmares. Hierarchical regression models showed that current severity of insomnia symptoms and the frequency and intensity of nightmares failed to predict suicidal thoughts and plans, beyond that explained by depressive symptoms, entrapment, and acquired capability for suicide. However, there were significant interaction effects between both entrapment and severity of insomnia, and entrapment and frequency and intensity of nightmares, in predicting suicidal thoughts and plans (Hochard et al., 2016). The methodological approach of this study was rated highly against the quality criteria, with prominent strengths including the use of power calculations to define the target sample size, validated measurement of study variables, and theoretically-driven, clearly defined, hypotheses. However, the analysis focusing on insomnia would have been strengthened by using nightmares as a control variable. Similarly, the analysis of nightmares would have been improved by the inclusion of insomnia as a control variable. These inclusions would have allowed the specific elements of sleep problems which amplify suicidal thoughts to be isolated.

##### 3.3.1.4 Summary of research examining cognitive appraisals

Collectively, there is strong evidence of the role of negative cognitive appraisals in the sleep/suicide relationship from six studies, of moderate (Littlewood et al., 2016, Woosley et al., 2014) to high quality (Bozzay et al., 2016, Hochard et al., 2016, Littlewood et al., 2016, McCall et al., 2013). However, it is important to note that there is considerable heterogeneity between these six studies, pertaining primarily to the measurement of different cognitive appraisals, and using different methodological approaches.

#### 3.3.2 Psychosocial factors

##### 3.3.2.1 Perceived burdensomeness, thwarted belongingness and social isolation

One of the most prominent contemporary models of suicide is the Interpersonal Theory of Suicide (IPTS; Joiner, 2005, Van Orden et al., 2010) which proposes that the simultaneous presence of two psychosocial factors of perceived burdensomeness and thwarted belongingness leads to desire for suicide. Perceived burdensomeness reflects both an individual's belief that they are a liability to others, coupled with perceptions of self-hatred (Van Orden et al., 2010). The ‘need to belong’ is said to be a fundamental human need (Baumeister & Leary, 1995) and in instances where this need is unmet, it is suggested that this gives rise to feelings of thwarted belongingness (Van Orden et al., 2010). Conceptually, both loneliness and the absence of reciprocally-caring relationships represent dimensions of thwarted belongingness. Four studies to date have directly tested the extent to which these psychosocial constructs from the IPTS can account for the relationship between sleep problems (insomnia and nightmares) and suicidality (Chu et al., 2016, Golding et al., 2015, Nadorff et al., 2014).

Nadorff et al. (2014) reported findings from two separate studies with different samples of healthy university students. In both studies, the frequency and intensity of nightmares significantly predicted suicidality, independent of perceived burdensomeness, thwarted belongingness and depressive symptoms. This indicates that the constructs defined within the IPTS do not fully explain the relationship between nightmares and suicidality. However, the evidence for the severity of insomnia symptoms was less clear. Findings from the second study were consistent with those reported for nightmares, in that the relationship between insomnia and suicidal behaviours was significant, independent of perceived burdensomeness, thwarted belongingness, and depressive symptoms. In contrast, in the first study the relationship between insomnia and suicidality was not significant when these three factors were added to the analysis model. These mixed findings suggest that the relationship between insomnia and suicidality may be mediated by the variables outlined within the IPTS but only under specific circumstances. For instance, the authors suggested that the duration of insomnia symptoms may drive the association between insomnia and suicidality in university samples (Nadorff, Nazem, & Fiske, 2013). Therefore, differences between patterns of results for study 1 and study 2 may stem from differences in the duration of insomnia experienced by the different samples (Nadorff et al., 2014). Based on the evaluation against the quality criteria, these two studies were assessed as being of a high methodological quality with the use of clear, theoretically based, research questions to be commended (Nadorff et al., 2014).

Members of the same research group recently conducted a similar study but with an older sample of adults aged 55 to 75 years (Golding et al., 2015). In addition to measuring the severity of insomnia symptoms and frequency and intensity of nightmares, the duration with which participants had experienced symptoms of insomnia and nightmares was also assessed. Using hierarchical regression analyses, perceived burdensomeness and thwarted belongingness were added into the first step of the model, followed by insomnia symptoms and frequency and severity of nightmares into the next step, and nightmare duration and insomnia duration were entered into the last step of the model. Consistent with the studies by Nadorff et al. (2014), nightmare duration and nightmare symptoms significantly predicted suicidality, independent of the IPTS constructs and insomnia. However, neither symptoms of insomnia nor insomnia duration significantly predicted suicidality, after controlling for the IPTS variables (Golding et al., 2015). This patterns of results are in line with those from Nadorff et al. (2014) study 1, and suggest that the IPTS variables may account for the insomnia/suicidality relationship. In extending the earlier work conducted by Nadorff et al. (2014), this later study demonstrated the same methodological rigour in testing theory-driven hypotheses, and simultaneously controlling for duration and symptoms of different types of sleep problems.

A study carried out in South Korea focused solely on the mediational role of thwarted belongingness, based on the empirical evidence that insomnia appears to be associated with increased feelings of loneliness (Chu et al., 2016). Accordingly, they conducted a cross-sectional questionnaire study with undergraduate students to examine the extent to which thwarted belongingness mediated the relationship between severity of insomnia symptoms and suicidal thoughts. As predicted, the relationship between insomnia severity and suicidal thoughts was mediated by thwarted belongingness (Chu et al., 2016).This research provides further evidence that the association between insomnia and suicidality may function via variables described by the IPTS, and specifically by thwarted belongingness (Chu et al., 2016, Golding et al., 2015, Nadorff et al., 2014). Merits of this study include the theoretical and empirically based hypotheses and utilisation of validated questionnaires. However, replication in clinical samples is necessary. The role of social isolation in explaining the link between sleep problems and suicidality was highlighted in the qualitative study included within this review (Littlewood, Gooding, Kyle, et al., 2016). The importance of social support in buffering suicidal thoughts and behaviours was emphasised by these participants, and consequently, social isolation was seen as contributing to suicidality. When participants were awake in the night they felt isolated from friends and family members. Seemingly, night-time acted as a barrier, preventing them from gaining social support and also fed into participants' sense of loneliness. Participants also recognised that isolation from social support provided an opportune time for suicide attempts, as there was a reduced chance of intervention from a family member or friend during the night-time. Social isolation was also acknowledged as a daytime consequence of poor sleep the previous evening. Participants subsequently felt irritable and had low energy, which both deterred them from seeking social interactions and threatened the continuation of social relationships.

##### 3.3.2.2 Summary of research examining psychosocial factors

In summary, the role of psychosocial factors in relation to the association between sleep problems and suicidality is unclear. Differences in findings between studies examining insomnia and nightmares indicate that the role of psychosocial factors may differ as a function of the specific type of sleep problem being experienced. For instance, consistently across three cross-sectional studies, the relationship between nightmares and suicidality operated independently of thwarted belongingness and perceived burdensomeness (Golding et al., 2015, Nadorff et al., 2014). In contrast, mixed findings indicated that social isolation or thwarted belongingness (Chu et al., 2016, Golding et al., 2015, Littlewood et al., 2016, Nadorff et al., 2014) and perceived burdensomeness (Nadorff et al., 2014) may partially account for the relationship between insomnia symptoms and duration, and suicidality. The five studies that examined psychosocial factors in the sleep/suicide relationship were all appraised as being of high quality.

#### 3.3.3 Emotion regulation strategies

##### 3.3.3.1 Rumination and emotional regulation

The current review identified one cross-sectional quantitative study which examined the interrelations between sleep quality, suicidality, and emotion regulation strategies, namely, rumination, cognitive reappraisal, and expressive suppression (Weis et al., 2015). In this study, rumination was conceptualised as a cognitive process, whilst in the wider literature rumination has been described as a maladaptive form of coping and emotion regulation (Aldao, Nolen-Hoeksema, & Schweizer, 2010). Conceptually, rumination can be described as repeatedly thinking about the causes and consequences of an individual's negative emotional state. Cognitive reappraisal refers to modifying the ways in which a stressor is evaluated. Expressive suppression reflects the ability to inhibit the outward expression of emotional states (Aldao et al., 2010). Cognitive reappraisal and expressive suppression were measured using the Emotion Regulation Questionnaire (Gross & John, 2003). Cognitive reappraisal is considered to be a more positive strategy and expressive suppression has been acknowledged to represent a maladaptive emotion regulation strategy (Aldao et al., 2010), although this may be culturally specific (Soto, Perez, Kim, Lee, & Minnick, 2011). Despite this, the authors chose not to analyse the two subscales separately, and instead performed analysis on the total scale score as an overall measure of emotion regulation. Preliminary analyses indicated which study variables predicted suicidality. Different models composed of the significant predictors of suicidality were compared. The best-fitting model indicated that the relationship between poor sleep quality and suicidality operated indirectly through rumination, depression, and emotional regulation as parallel mediators. Furthermore, the relationship between emotional regulation and suicidality also functioned indirectly through depression, and rumination. Methodologically, a clear strength of the study by Weis et al. (2015) was the use of validated scales to measure all study variables. Although this study provided evidence to support the role of rumination within the sleep quality/suicide relationship, the specific roles of cognitive reappraisal and expressive suppression were unclear. In addition, the failure to provide a rationale for the sample size and the absence of important statistical information, such as confidence intervals, were reflected in the moderate quality rating (see Table 1).

Finally, in the only qualitative study, disturbed sleep contributed to rumination because participants felt less able to distract themselves from negative, repetitive, thought processes (Littlewood, Gooding, Kyle, et al., 2016). However, it was not clear from participant's quotes or supporting narratives whether individuals were describing rumination or a form of more general negative thinking. Consequently, implications of these findings are limited.

##### 3.3.3.2. Summary of research examining emotion regulation strategies

Two studies included within this review provided tentative evidence that emotion regulation strategies may partially account for the association between sleep problems and suicidality (Littlewood et al., 2016, Weis et al., 2015). However, conclusions from these findings must be tempered because they are based on only two studies, and the qualitative study failed to provide data which clearly depicted rumination. Further, research has yet to examine the individual contribution of different types of emotion regulation strategies to sleep/suicide relationships.

#### 3.3.4 Risk behaviours

##### 3.3.4.1 Acquired capability for suicide

Whilst two psychosocial constructs of the IPTS, that is, perceived burdensomeness and thwarted belongingness, are said to trigger the desire for death by suicide, the third construct, ‘acquired capability for suicide’, is posited to account for the transition from suicidal desire to making suicide attempts (Joiner, 2005, Van Orden et al., 2010). Here, both a lowered fear of death and an elevated tolerance for physical pain are purported to develop via habituation in response to repeated exposure to painful and life-threatening or fear-inducing experiences (Van Orden et al., 2010). Acquired capability for suicide was investigated by four studies in the context of the sleep/suicide relationship.

Three studies, published in two papers, conducted by members of the same research group, sought to examine whether the relationship between sleep problems and suicidality could be accounted for by the IPTS (Golding et al., 2015, Nadorff et al., 2014). Collectively, findings from these studies indicated that sleep problems were related to suicidality, independent of acquired capability for suicide (Golding et al., 2015, Nadorff et al., 2014). Hochard et al. (2016) proposed alternative hypotheses whereby acquired capability was posited to interact with both severity of insomnia symptoms and the frequency and intensity of nightmares, to predict suicidal thoughts. Indeed, hierarchical regression models confirmed these predictions, showing that the interactions between both acquired capability and insomnia symptoms, and acquired capability and nightmares, accounted for additional variance in suicidal thoughts beyond the effect of depressive symptoms (Hochard et al., 2016). In this study, acquired capability was operationalised as deliberate self-harm which is less precise compared to the conceptualisation used by Joiner and colleagues (Joiner, 2005, Van Orden et al., 2010). Whilst frequency of self-harm has been shown to predict greater levels of acquired capability for suicide (Willoughby, Heffer, & Hamza, 2015), this is not the sole mechanism by which people develop the capability for suicide (Van Orden et al., 2010). Therefore, replication of these findings using The Acquired Capability for Suicide Scale (Van Orden, Witte, Gordon, Bender, & Joiner, 2008) is warranted.

##### 3.3.4.2 Aggressive behaviour

A hypothesised mediational pathway was tested by Zschoche and Schlarb (2015) who predicted that the relationship between sleep quality and suicidality would operate indirectly through aggressive behaviour and depression. A sample of adolescents aged between 14 and 18 years completed self-report measures of sleep quality, suicidality, aggressive behaviour, and depression. The mediational model indicated that the relationship between sleep quality and suicidality was partially mediated by depression, but not aggressive behaviour. However, the direct relationship between sleep quality and suicidality remained significant.

##### 3.3.4.3 Summary of research examining risk behaviours

Four of the identified studies suggest that the relationship between sleep problems and suicidality is unlikely to function via acquired capability for suicide (Golding et al., 2015, Nadorff et al., 2014) nor aggressive behaviour (Zschoche & Schlarb, 2015). The fifth study took an alternative approach to show that the interaction between sleep problems and acquired capability predicted increased suicidal thoughts (Hochard et al., 2016). Although the quality of this evidence was rated as moderate (Zschoche & Schlarb, 2015) to high (Golding et al., 2015, Nadorff et al., 2014), given that this divergent evidence is based on a small number of studies all of which were conducted with non-clinical samples, it would be inappropriate to discount the role of risk behaviours at this point.

## 4 Discussion

A clear, and impactful, finding of this systematic review was that the empirical literature examining the role of psychological factors that underpin the relationship between sleep problems and suicidality is in its infancy. An important aspect of this review was the critical evaluation of the evidence against six criteria quantifying the quality of the methodology and analyses used by each of the studies. Whilst all of the studies were judged to be of moderate to high quality, it is important to remember that this is based on quality criteria specific to cross-sectional and qualitative designs. The predominant use of cross-sectional designs limits interpretation of the directionality of posited pathways. Although mediational analyses are commonly conducted within cross-sectional designs, it should be noted that no temporal or causal relationships can be inferred from this data (Winer et al., 2016). Rather, studies evaluated by this review utilised mediational analyses to account for the shared relationships between psychological factors, sleep problems, and suicidal thoughts and behaviours. These findings provide the groundwork from which alternative designs, such as prospective, longitudinal, experience sampling, and experimental methods, can examine the putative pathways highlighted by this review.

The identified literature solely focused on subjective measures of sleep, and is yet to examine specific complexities of sleep, such as sleep stages, continuity and circadian patterning, and hence would benefit from objective measurement of sleep via polysomnography or actigraphy. More broadly, objective sleep disturbance has been shown to be associated with both suicidal thoughts and behaviours (Agargun and Cartwright, 2003, Ballard et al., 2016, Keshavan et al., 1994, Sabo et al., 1991) and mental health problems (Baglioni et al., 2016). Quantitative research would benefit from measuring different parameters of sleep and sleep problems, and from including adjustments in data analyses to establish the independent contribution of each of the different facets of sleep (e.g., continuity, quality) in conferring risk for suicidal thoughts and behaviours. Furthermore, given the growing evidence showing that people who experience suicidal thoughts also report suicidal imagery (Hales, Deeprose, Goodwin, & Holmes, 2011), future research should examine the relationship between nightmare and dream content in relation to suicidal thoughts and behaviour. It is possible that suicidal imagery during sleep may trigger or intensify perceptions of entrapment or hopelessness for an individual, due to the uncontrollable nature of dream and nightmare content.

Although research studies have reported significant associations between suicidality and a range of sleep problems, including insomnia, nightmares, and poor sleep quality (Bernert et al., 2015, Malik et al., 2014, Pigeon et al., 2012), evidence from this review suggests that the psychological factors which underpin these relationships may differ as a function of the specific sleep problem which is being experienced (Golding et al., 2015, McCall et al., 2013, Nadorff et al., 2014). However, findings from the only qualitative study included in this review described core pathways thought to underpin the relationship between suicidality and different types of sleep problems, including social isolation, defeat and entrapment (Littlewood, Gooding, Kyle, et al., 2016). Future empirical work should seek to ascertain whether the identified psychological factors represent core, transdiagnostic, mechanisms which underpin the sleep/suicide relationship.

Related to this, it is important for work in this area to extend the current linear, unidirectional focus predominantly taken to investigate bi-directional pathways between sleep problems, suicidality and psychological factors. The negative daytime consequences of sleep problems have been postulated to have an adverse effect on sleep quality the following night, (Harvey, 2008). Hence, future studies should examine, not only the effects of poor sleep on suicidal thoughts, but also the degree to which suicidal thinking affects quality of sleep.

Regardless of the methodological issues inherent in the identified literature, there is sufficient initial evidence to indicate that negative cognitive appraisals, social isolation, and unhelpful coping and emotion regulation strategies may partially account for the sleep/suicide relationship. Furthermore, it is reassuring that the quality of the methodology and analytical approach taken within the reviewed studies were rated as moderate to high. Notably, ten of the eleven studies sought to quantify the role of depression when examining interrelationships between sleep, suicidal behaviour and psychological factors. Future research should establish the extent to which aspects of specific mental health problems may moderate the role of identified psychological factors in the context of the sleep/suicide relationship.

**Fig. 2:**
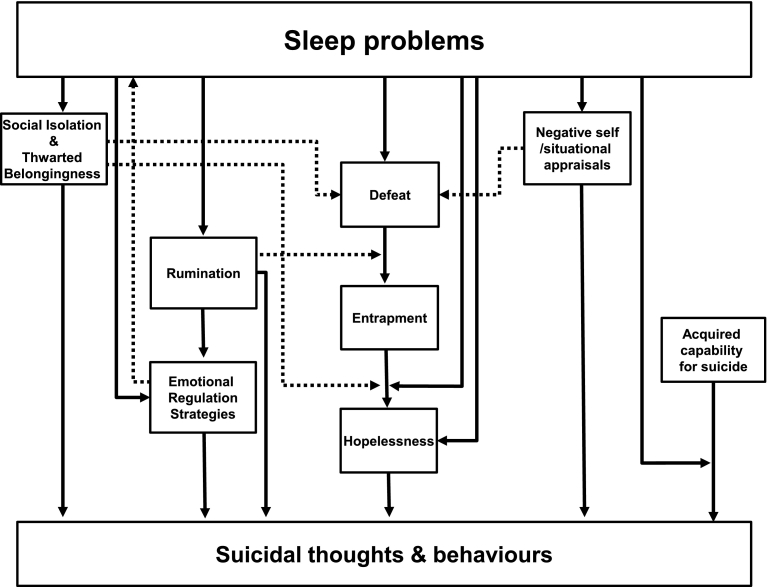
Solid lines depict mediational or moderational pathways from the review findings, and dotted lines indicate additional pathways as predicted by contemporary models of suicide.

It is important to integrate the findings of this review with contemporary models of suicidal thoughts, plans and behaviours so that these models can be further developed and tested in the context of sleep disorders. A visual schematic diagram of the integrated theoretical and empirical findings are presented in Fig. 2. This figure represents the pathways largely using causal, unidirectional, pathways based on the interpretation provided in the literature. As all of the studies included in this review used cross-sectional designs it will be important in future studies to test bi-directional, cyclical, temporal, and causal relationships. For instance, it is possible that suicidal thinking may delay sleep onset, and hence cause or reinforce sleep disturbance.

### 4.1 The role of psychological processes in the sleep/suicidality relationship

#### 4.1.1 Cognitive appraisals

Taken together, empirical findings from six studies suggested that cognitive appraisals may play a key role in explaining the relationship between sleep problems and suicidal thoughts and behaviours. Specifically, four multi-step pathways could be identified from the review which incorporated five types of negative cognitive appraisals, namely, hopelessness, defeat, entrapment, and situational and self-appraisals (see Fig. 2).

First, a two-step pathway can be posited, whereby sleep problems trigger hopelessness, which in turn leads to suicidal thoughts (Woosley et al., 2014). Hopelessness has been shown to be one of the strongest predictors of suicidal thoughts and behaviours (O'Connor & Nock, 2014). However, empirical evidence implies that hopelessness does not fully explain the relationship between sleep problems and suicidal thoughts and behaviours (Ribeiro et al., 2012, Winsler et al., 2015). Alternatively, hopelessness may operate alongside other mechanisms to explain the link between sleep and suicidality (Bozzay et al., 2016, Littlewood et al., 2016).

Second, a significant four-step mediational pathway was reported whereby nightmares led to perceptions of defeat, entrapment, hopelessness and finally to suicidal thoughts and behaviour (Littlewood, Gooding, Panagioti, et al., 2016). The central role of defeat and entrapment in suicidality is emphasised within contemporary theoretical models of suicidal thoughts and behaviours (Johnson et al., 2008, O'Connor, 2011, Williams and Williams, 1997, Williams et al., 2005) and the broader empirical literature (Siddaway et al., 2015). In addition, theoretical and empirical evidence suggests it may be prudent to expand work relating to defeat to examine the role of inferiority (Gilbert and Allan, 1998, Lee et al., 2010). Theoretically, inferiority is posited to emerge from perceptions of having a low social ranking in comparison to others (Gilbert & Allan, 1998). Data from a large cross-sectional study indicated that inferiority predicts suicidal thoughts, independent of insomnia, depression, anxiety, unemployment and hostility (Lee et al., 2010). Empirically, conceptual commonalities and divergences between perceptions of inferiority and defeat in relation to suicidal thoughts and behaviours have yet to be identified.

The third pathway reflects that sleep problems, such as insomnia or nightmares, may contribute to increased suicidal thoughts via their interaction with perceptions of entrapment (Hochard et al., 2016). In this sense, nightmares and insomnia are posited to act as moderators, intensifying the association between entrapment and suicidality.

Fourth, a pathway was identified from sleep problems to negative situational and self-appraisals, which in turn were associated with suicidal thoughts and behaviours (Littlewood et al., 2016, McCall et al., 2013). This is consistent with the Schematic Appraisals Model of Suicide (SAMS;Johnson et al., 2008) which posits that negative situational and self-appraisals are particularly deleterious within suicide pathways, as both can trigger perceptions of defeat and entrapment, from which suicidal thoughts are posited to emerge (see Fig. 2). Specifically, perceptions of defeat and entrapment were shown to mediate the relationship between self-appraisals of emotional-coping and social problem-solving ability, and suicidal behaviour in individuals who had experienced trauma (Panagioti, Gooding, Taylor, & Tarrier, 2012). Although both situational and self-appraisals are broad concepts, proponents of the SAMS highlight the specific relevance of three types of self-appraisals, namely, evaluations of personal attributes (such as self-esteem), cognitive-emotional abilities (such as perceptions of social problem-solving and emotional coping) and the perceived ability to draw upon social support (Johnson et al., 2008).

Indeed, evidence for the fifth pathway encompassed appraisals of social problem-solving, and suggested that insomnia led to perceptions of fatigue, which contributed to negative appraisals of social problem-solving, which triggered perceptions of hopelessness, from which suicidal thoughts emerged (Bozzay et al., 2016). The pathways which have been identified represent an excellent starting point but, they now must be tested with the goal of establishing convergent evidence generated from the use of different methodological approaches. Future research would benefit from investigating the ways in which sleep problems interact with the specific appraisals suggested as being key to pathways to suicidal thoughts and behaviours (see Fig. 2).

#### 4.1.2 Psychosocial factors

Convergent qualitative and quantitative results have shown that social isolation or thwarted belongingness may account for the relationship between sleep and suicidal thoughts and behaviours (see Fig. 2; Chu et al., 2016, Golding et al., 2015, Littlewood et al., 2016, Nadorff et al., 2014). This is in agreement with findings from the broader literature. For example, people with insomnia reportedly feel isolated due to lack of understanding from friends or family, disengage from social activities due to fatigue, and fail to seek social support (Henry et al., 2013, Kyle et al., 2010). Furthermore, social isolation has been identified as one of the strongest predictors of suicidal thoughts and behaviours, and is said to be indicative of thwarted belongingness (Van Orden et al., 2010). However, there was divergent data from four cross-sectional studies (Chu et al., 2016, Golding et al., 2015, Nadorff et al., 2014), with three providing evidence to suggest that thwarted belongingness may account for the insomnia/suicidality relationship (Chu et al., 2016, Golding et al., 2015, Nadorff et al., 2014). In contrast, Nadorff et al.'s (2014) study 2 reported a significant association between insomnia and suicidality, beyond thwarted belongingness. The authors provided one possible explanation for the mixed quantitative findings, speculating that sample differences between duration of insomnia symptoms may account for the divergent pattern of results (Nadorff et al., 2014). Indeed, previous research has indicated that duration of insomnia is associated with suicidality in university students, independent of insomnia symptoms (Nadorff, Nazem, et al., 2013). This possibility should be examined to quantify the extent to which the interrelationships between insomnia, suicidality, social isolation and thwarted belongingness, vary as a function of duration of insomnia.

From the perspective of the SAMS, social isolation can be understood as a negative appraisal of social support, and hence, is posited to be indirectly related to suicidal thoughts and behaviours via perceptions of defeat and entrapment (see Fig. 2; Johnson et al., 2008, Taylor et al., 2010). Alternatively, the Integrated Motivational-Volitional model (IMV) postulates that negative appraisals of social support interact with entrapment to predict suicidal thoughts (see Fig. 2; O'Connor, 2011). These hypotheses should be examined with different types of path analyses, such as moderated mediational modelling, to develop a greater understanding of the role of social isolation in the sleep problem/suicide relationship.

#### 4.1.3 Emotion regulation strategies

Three pathways could be identified from this review which incorporate the emotion regulation strategies of rumination, cognitive reappraisals, and expressive suppression see Fig. 2 (Littlewood et al., 2016, Weis et al., 2015). There was convergent, albeit tentative, evidence from two studies (Littlewood et al., 2016, Weis et al., 2015) regarding a possible mediating role of rumination, in the relationship between sleep problems and suicidality. This is bolstered by the wider literature which shows that pre-sleep rumination is associated with delayed sleep onset (Pillai, Steenburg, Ciesla, Roth, & Drake, 2014), and that rumination is associated with suicidal thoughts and behaviours (Morrison & O'Connor, 2008). Furthermore, repeatedly thinking about suicide may prevent healthy sleep and also contribute to vital exhaustion (Kerkhof & van Spijker, 2011). Subsequently, the failure to escape suicidal thoughts via sleep may then reinforce and intensify suicidal thinking. Theoretically, the IMV postulates that rumination acts as a moderator in suicidal pathways, whereby ruminative processes strengthen the relationship between defeat and entrapment (see Fig. 2; Dhingra et al., 2016, O'Connor, 2011). This should be tested further with a moderated-mediational model to examine whether rumination acts as a mediator of the relationship between sleep problems and suicidality, or as a moderator of the relationship between defeat and entrapment.

Two further significant pathways were reported by Weis et al. (2015). First, the association between sleep problems and suicidality operated indirectly through cognitive reappraisal and expressive suppression. Second, a significant three-step pathway indicated that the association between sleep problems and suicidality operated indirectly, first, through cognitive reappraisal and expressive suppression, which were considered together, then to rumination (Weis et al., 2015). Consistent with this, poor sleepers have found it more difficult to implement cognitive reappraisal strategies than good sleepers (Mauss, Troy, & LeBourgeois, 2013). Additional studies should seek to examine the separate contribution of these two types of emotion regulation strategies, given that cognitive reappraisal is considered to be an adaptive process which is protective of mental health problems, whilst expressive suppression may be viewed as maladaptive (Aldao et al., 2010), and associated with greater levels of mental health problems in some contexts (Soto et al., 2011). Furthermore, it is important to acknowledge the possible bidirectional relationship between emotional dysregulation and sleep disturbance (Harvey, Murray, Chandler, & Soehner, 2011). A challenge for future research projects is to develop ways of rigorously testing unidirectional and bidirectional pathways involving sleep, emotional regulation, and suicidality.

#### 4.1.4 Risk behaviours

Research examining the role of risk behaviours in relation to sleep problems and suicidal thoughts and behaviours has thus far examined ‘acquired capability for suicide’ (Golding et al., 2015, Hochard et al., 2016, Nadorff et al., 2014) and aggressive behaviour (Zschoche & Schlarb, 2015). There was mixed evidence to support a role of acquired capability for suicide in accounting for the relationship between sleep problems and suicidality. Three studies reported non-significant findings (Golding et al., 2015, Nadorff et al., 2014) and a single study (Hochard et al., 2016) showed significant interaction effects between insomnia and acquired capability, and nightmares and acquired capability, in relation to suicidal thoughts, albeit using a measure of deliberate self-harm as an indicator of acquired capability, as opposed to a scale constructed to measure this construct. However, the findings of Hochard et al. (2016) fit with recent cross-sectional and longitudinal studies which indicated that acquired capability for suicide significantly interacted with states of hyperarousal,^2^ such as sleep disturbance, to predict both suicidal thoughts (Ribeiro, Silva, & Joiner, 2014) and death by suicide (Ribeiro, Yen, Joiner, & Siegler, 2015), independent of depression. Specifically, hyperarousal amplified suicidal thoughts and risk in individuals with high levels of acquired capability for suicide. However, there was no interaction between low capability for suicide and hyperarousal. In order to advance our understanding in this area, subsequent work should seek to examine the specific independent moderating effects of sleep problems, as a form of hyperarousal, on the relationship between acquired capability for suicide and suicidality (see Fig. 2).

Evidence from the single study included in this review reported that aggressive behaviour did not account for the relationship between sleep problems and suicidality (Zschoche & Schlarb, 2015). This is surprising as sleep problems are a risk factor for aggressive behaviour (Kamphuis, Meerlo, Koolhaas, & Lancel, 2012) and aggression has been associated with suicidal behaviours (Gvion & Apter, 2011). Aggressive behaviour only reflects one facet of aggression. Consequently, examination of the role of different aspects of aggression relative to the sleep/suicide relationship is warranted. For instance, hostility may reflect feelings of irritability or anger, but may not necessarily result in aggressive behaviour. Hostility has been associated with a greater risk of suicidal thoughts and behaviours (Ferraz et al., 2013, Jeon et al., 2013, Zhang et al., 2012), and was found to predict suicidal ideation, independent of insomnia (Lee et al., 2010).

### 4.2 Clinical implications

This review highlights the need to collect further evidence to facilitate the future development of effective clinical interventions (Craig et al., 2008). Whilst it would be premature to make suggestions concerning the directions that such interventions should take, four implications for clinical practice are evident. First, when working with clients exhibiting suicidal thoughts and behaviours it is important to assess and monitor co-occurring sleep problems using measures and scales with established psychometric properties. Second, our review suggests that restoration of healthy sleep could be beneficial to suicidal clients, particularly in conjunction with interventions targeted at resolving negative cognitive appraisals. Third, the present studies highlighted interrelations between sleep problems, social isolation and suicidality (Chu et al., 2016, Golding et al., 2015, Littlewood et al., 2016, Nadorff et al., 2014). Establishing access to social support both during day-time and night-time hours may help to reduce vulnerability for suicidal thoughts and behaviours. Fourth, comorbid sleep problems increased suicidality in individuals who displayed high capability for suicide (Hochard et al., 2016). Consequently, improving the sleep of this high-risk group may reduce vulnerability to suicide in the short-term, and permit therapeutic techniques to be used more effectively, when a greater attentional load is placed upon clients' cognitive-evaluative skills (Tarrier et al., 2013). In terms of appropriate sleep interventions, cognitive behavioural therapy for insomnia has been shown to effectively reduce insomnia symptoms (Wu et al., 2015), and suicidal thoughts (Trockel et al., 2015). No comparative studies have been conducted to examine the impact of nightmare-targeted treatments on co-occurring suicidality.

### 4.3 Strengths and limitations of the review

There were four major strengths of the current review. First, one of the most important strengths is the novel theoretical contribution by integrating initial empirical findings with contemporary psychological models of suicide to identify ways in which these models can be optimally developed to provide explanations of the relationship between sleep problems and suicidal thoughts and behaviours. It also illustrates where predictions differ across these psychological models, and indicates ways in which differentiable hypotheses can be generated. Second, the systematic search strategy was informed by the PRISMA statement, and comprehensive search terms were used which encompassed both text and MeSH terms that were customised for use with each of four major bibliographic databases. Third, existing tools which evaluate the methodological quality of studies were adapted to enable the systematic appraisal of quantitative and qualitative papers against six comparable quality criteria. Fourth, the last author conducted reliability checks to ensure rigour of both the screening process and the critical evaluation of the methodological quality of the identified studies. Hence, this review makes a number of important theoretical and clinical contributions, and used robust procedures to ensure methodological rigour.

Three limitations of the review should be considered. First, intentionally inclusive sampling criteria resulted in the inclusion of studies with wide ranging samples, covering healthy, clinical, adolescent and adult populations. It is important for future work to ascertain the extent to which the proposed pathways can account for the sleep/suicide relationship across different clinical and non-clinical populations. Second, the inclusion criteria were restricted to studies published in English-language, peer-reviewed journals. This may have limited the results by excluding the grey literature (e.g., Department of Health reports in the UK) or those not published in English. That said, one clear benefit of the peer-review process is that it acts as a quality control mechanism (Campanario, 1998). Furthermore, it remains challenging to systematically search literature published outside of peer-reviewed journals which are not routinely included within all bibliographic resources (Bellefontaine & Lee, 2014). Third, review findings were integrated with psychological theory to develop a research agenda to guide future empirical investigation. Whilst outside of the scope of the current review, it may also prove fruitful to examine the evidence base for psychological factors which have established associations with i) sleep problems, and ii) suicidal behaviours, but which have not yet been investigated in the context of the sleep/suicide relationship.

## 5 Conclusion

It is clear that sleep problems are related to suicidal thoughts and behaviours. However, research examining the psychological processes that may underpin this relationship is at an early stage. Preliminary evidence suggests that the relationship between sleep problems and suicidal thoughts and behaviours may function via three types of psychological factors which are negative cognitive appraisals (Bozzay et al., 2016, Littlewood et al., 2016, McCall et al., 2013, Woosley et al., 2014), social isolation and thwarted belongingness (Chu et al., 2016, Golding et al., 2015, Littlewood et al., 2016, Nadorff et al., 2014), and emotion regulation strategies (Weis et al., 2015, Littlewood et al., 2016). Additionally, a single study reported an interaction effect between sleep problems and acquired capability for suicide in relation to suicidality (Hochard et al., 2016). Theoretically, this resonates with elements of four contemporary models of suicidal behaviour, the IPTS (Joiner, 2005, Van Orden et al., 2010), the IMV (O'Connor, 2011), and the SAMS (Johnson et al., 2008). Furthermore, integration of the review findings with relevant aspects from these contemporary theories, allowed the development of a clear research agenda (see Fig. 2) from which longitudinal, experience sampling, and experimental designs should be utilised to generate convergent evidence. Clinically, investigating the role of psychological processes in pathways which link sleep problems and suicidality is fundamental to the development of suicide prevention interventions. Whilst it would be premature to suggest specific interventions, it would seem prudent for clinicians to consider evaluating and managing sleep problems in the context of suicidality.

## Figures and Tables

**Table:** 

Database	Suicide MeSH terms	Sleep MeSH terms
PsycINFO	suicide OR suicide prevention OR suicidal ideation OR attempted suicide OR suicid* OR parasuicide OR self destructive behaviour OR self*harm* OR NSSI* OR self injurious behaviour OR self inflicted wounds OR self mutilation OR DSH* OR drug overdoses OR self poison* OR overdose* OR hopelessness	sleep OR sleep disorders OR sleep deprivation OR sleep apnea OR sleep talking OR insomnia OR dyssomnia OR narcolepsy OR hypersomnia OR sleepiness OR parasomnias OR sleepwalking OR nightmares OR night terror*
EMBASE	suicide OR suicid* OR suicidal behaviour OR suicide attempt OR parasuicid* OR suicidal ideation OR self*harm* OR self*injur* OR self-injury OR Self Injurious Behavior OR NSSI* automutilation OR DSH* OR drug overdose OR self poisoning OR self*poison* OR self*mutilat* OR self*destruct* OR self*inflict* OR overdose OR hopelessness	sleep OR circadian rhythm sleep disorder OR sleep disorder OR central sleep apnea syndrome OR sleep deprivation OR “International Classification of Sleep Disorders” OR sleep disordered breathing OR sleep quality OR sleep pattern OR sleep time OR insomnia OR primary insomnia OR narcolepsy OR hypersomnia OR sleep walking OR nightmare OR parasomnia OR night terror*
MEDLINE	suicidal OR suicidal ideation OR suicide, attempted OR suicid* OR parasuicid* OR self-injurious behaviour OR self*harm* OR self-injur* OR self-inflict* OR poisoning OR drug overdose OR self-poison* OR overdose OR NSSI* OR DSH* OR self mutilat* OR hopelessness	sleep OR sleep disorders OR dyssomnias OR sleep deprivation OR circadian rhythm OR parasomnias OR insomnia OR insomnia, fatal familial OR hypersomnia* OR night terrors OR nightmare* OR narcolepsy
Web of Science	suicid* OR self*harm* OR self-injur* OR self*injur* OR NSSI* OR DSH OR self*poison* OR self*destruct* OR overdose OR hopelessness	sleep* OR sleep disorders* OR sleep deprivation OR sleep apnea OR insomnia* OR dyssomnia* OR narcolepsy OR hypersomnia* OR sleepiness OR parasomnia* OR sleepwalking OR night terror* OR nightmare*

**Table:** 

Criterion	Rating for quantitative studies	Rating for qualitative studies
Methodology (maximum of 6)		
Research question (RQ), aims and design	2 = Clear RQ/aims that were related to relevant theoretical frameworks, and an appropriate design. 1 = Clear RQ/aims with appropriate design. 0 = RQ/aims are not clear and/or design is not appropriate for RQ/aims.	2 = Clear RQ/aim which is appropriate for qualitative method selected and purpose made explicit, such as descriptive/explanatory intent, theory building, hypothesis testing. 1 = Clear RQ/aim which is appropriate for qualitative method selected. 0 = RQ/aims are not clear and/or method is not appropriate for RQ/aims.
Sampling method	1 = Appropriate for design. 0 = Not appropriate.	1 = Appropriate for design. 0 = Not appropriate.
Sample size	1 = Justified and satisfactory. 0 = Not justified.	1 = Justified. 0 = Not justified.
Data collection	2 = Data collected using validated measurement tools throughout. 1 = Some use of validated tools, or non-validated measurement tools, but tools are available or described. 0 = No description of the measurement tool.	2 = Justified and clearly outlined (e.g., for interviews- how were they conducted? Who conducted them? Were they structured/semi-structured/un-structured? Was a topic guide used? Were they audio-recorded?) Triangulation of data sources if appropriate. 1 = Partly justified and outlined. 0 = Not clear.
Analysis (maximum of 3)		
Reliability of analyses	2 = The study statistically controls for the most important and additional relevant confounding variables. 1 = The study statistically controls for the most important confounding variable (depression or other sleep problem). 0 = No control for confounding variables.	2 = Strategies to improve rigour of analyses as appropriate to outlined approach (i.e., deviant case analysis, inter-rater reliability, triangulation, member-checking) and analysis process is clearly documented. 1 = Analysis process is clearly documented and could be replicated. 0 = Insufficient detail regarding the analysis process.
Analysis is appropriate for RQ and aims	1 = Statistical test used to analyse the data is suitable and clearly described. Data reported in appropriate detail for the given statistical test, e.g., confidence intervals, probability level (p value). 0 = The statistical test is not appropriate, not clearly described or incomplete.	1 = Analysis is supported by sufficient data excerpts from a wide range of participants. 0 = Analysis is not well supported by data excerpts and/or there is an overreliance on specific participants.

## Footnotes

1 Nadorff et al., 2014 presents results from two independent studies.

2 Studies excluded from review because sleep disturbance was operationalised as part of overarousal, hence it was not possible to isolate relationships specific to sleep.
